# A mimic of tachycardia‐bradycardia syndrome in a patient with long‐standing persistent atrial fibrillation

**DOI:** 10.1002/ccr3.2058

**Published:** 2019-02-19

**Authors:** Akinori Sairaku, Takeshi Matsumoto, Hiroki Kinoshita, Hiroya Matsumura, Naoto Oguri, Nobuyuki Morishima

**Affiliations:** ^1^ Department of Cardiology, Cardiovascular Center Onomichi General Hospital Onomichi Japan

**Keywords:** long‐standing persistent atrial fibrillation, narrow QRS regular tachycardia, pause

## Abstract

Pause following incessant tachycardia is often encountered in clinical practice. We encountered a rare arrhythmic condition mimicking tachycardia‐bradycardia syndrome. We hereby describe the step‐by‐step diagnostic process.

## 1 CASE SUMMARY

**Figure 1:**
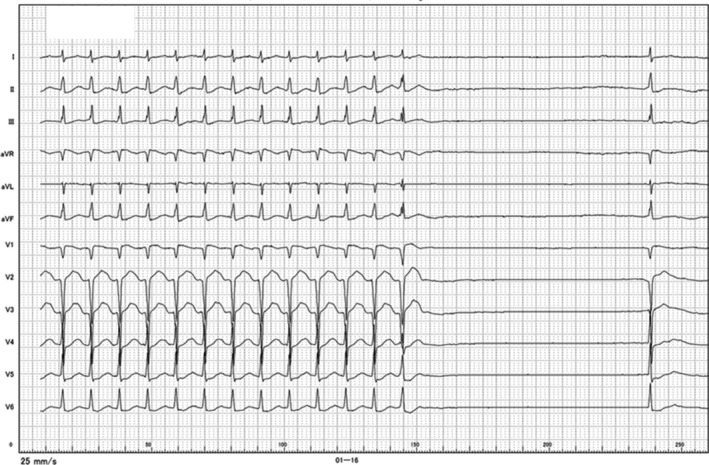
A 12‐lead electrocardiogram of the clinical arrhythmia. An incessant regular narrow QRS tachycardia with a heart rate of 140 beats/min following a 3.7‐s pause was noted

A 79‐year‐old man on regular hemodialysis who received aortic valve replacement 20 years prior was referred to our hospital complaining of palpitation and faintness. An electrocardiographic (ECG) documentation showed a regular narrow QRS complex tachycardia with a heart rate of 140‐150 beats/min, followed by 2‐4 second of pauses after its spontaneous termination (Figure 1). A series of tachycardia‐bradycardia events were consistently seen during the ECG monitoring in the emergency department. He was diagnosed with persistent atrial fibrillation (AF) 10 years prior, and since then, sinus conversion had not been documented. Although his left ventricular function was normal, his left and right atriums were largely enlarged upon echocardiographic examination. As is often the case with a long AF history and enlarged atrium, his fibrillatory waves were unclear.^1^ Is tachycardia‐bradycardia syndrome possible in a subject with long‐standing persistent AF?

## 2 STEP 1

**Figure 2:**
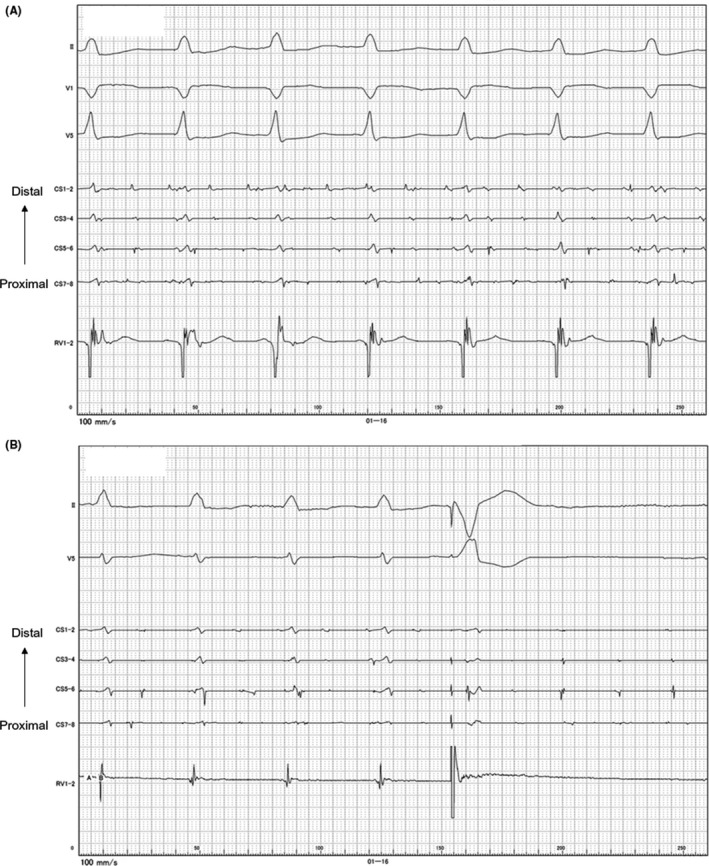
Intracardiac electrocardiograms illustrating the clinical tachycardia. Note that the right ventricular activation intervals are exactly constant while the atrial electrical activations recorded on an electrode catheter placed in the coronary sinus show that atrial fibrillation persists (A). A single extra‐stimulus from the right ventricle terminated the series of rapid ventricular electrical excitations (B). CS, coronary sinus; RV, right ventricle

An invasive electrophysiological study was carried out on the same day. Electrode catheters were inserted into the coronary sinus and right ventricle. We found that AF persisted not only during the pauses, but also during the tachycardia (Figure 2A), excluding tachycardia‐bradycardia syndrome. Another likely diagnosis was rapid ventricular response during AF with apparently regular RR intervals. If so, “pause during AF”^2^ could account for the prolonged RR interval following the tachycardia termination because it is often observed even in patients with rapid persistent AF.

## 3 STEP 2

We tested the possibility of rapid regular ventricular response during AF. Intravenous administration of isoproterenol turned the incessant tachycardia into a sustained one. We then gave him a bolus injection of 20 mg of adenosine triphosphate. The RR intervals, however, never prolonged during the tachycardia. A 100 mg verapamil injection also did not change them at all. As a result, atrioventricular dissociation during the tachycardia was revealed. We also found that ventricular pacing reproducibly terminated the tachycardia (Figure 2B). Was the tachycardia a ventricular tachycardia during AF?

## 4 STEP 3

The tachycardia was not sensitive to either an injection of 15 mg of nifekalant or 100 mg of lidocaine. Moreover, the morphology of the QRS complex during the tachycardia was exactly the same as that recorded during the absence of the tachycardia. Thus, a ventricular tachycardia during AF was ruled out. What was the final diagnosis? What was the proper treatment?

## 5 STEP 4

He was implanted with a pacemaker the day after the electrophysiological study, and then, 2.5 mg of bisoprolol was started. The tachycardia was successfully suppressed with those treatments. We finally diagnosed the tachycardia as junctional ectopic tachycardia during AF. What was the mechanism?

## 6 COMMENTARY

Junctional ectopic tachycardia is occasionally seen in pediatric patients who previously received cardiac surgery. However, it is rare in adults.^3^ Further, junctional ectopic tachycardia occurring during AF has not previously been reported, probably because it could occur only when certain rare conditions are fulfilled.

**Figure 3:**
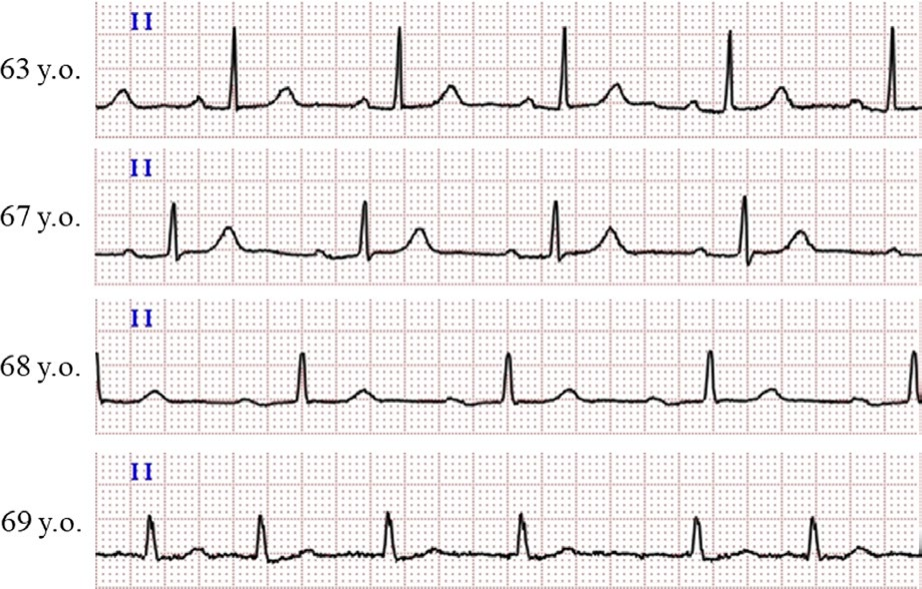
Electrocardiogram strips showing the chronological change in the PR interval. The PR interval prolonged over the years until the patient developed persistent atrial fibrillation at an age of 69 y old

The mechanism behind the tachycardia is considered to be abnormal automaticity arising from the atrioventricular junction.^3^ A single ventricular stimulation terminated the tachycardia in the present case, suggesting that an electrical excitation reaching the atrioventricular junction could stop the tachycardia if it was given during the excitation gap. Considering the tachycardia cycle length was around 400 ms, the AF cycle length was short enough to provide repetitive chances for the atrial impulses to irrupt into the excitation gap of the nodal tissue during the tachycardia. It is known that the number of atrial electrical excitations that transverse the atrioventricular node is limited during AF. Yet, even those that fail to penetrate through the node have a certain influence on the refractoriness of the nodal tissue, consequently suppressing the automaticity or reentrant involving the atrioventricular junction. This theory is called “concealed conduction”.^4^ Nevertheless, the tachycardia easily sustained in the present case. Why was that? Figure 3 illustrates the chronological change in the ECG. His atrioventricular conductivity had deteriorated over time. The long pause following the tachycardia termination could be regarded as evidence of his atrioventricular conduction disturbance. It may have largely prevented atrial impulses from reaching the node, probably making the electrical firings from the atrioventricular junction sustainable even during AF.

Junctional ectopic tachycardia is thought to be the result of an injury to the atrioventricular node‐His bundle complex.^5^ Therefore, the advanced damage to the atrioventricular junction itself may have played an important role in the development of the rare phenomenon.

## CONFLICT OF INTEREST

None declared.

## AUTHOR CONTRIBUTION

AS: involved in the invasive electrophysiological study, data analysis, and drafting the manuscript; HK, HM, and NO: involved in the invasive electrophysiological study and data analysis; TM and NM involved in the manuscript review.
